# CT radiomics combined with clinical and radiological factors predict hematoma expansion in hypertensive intracerebral hemorrhage

**DOI:** 10.1007/s00330-024-10921-2

**Published:** 2024-07-11

**Authors:** Fei Yu, Mingguang Yang, Cheng He, Yanli Yang, Ying Peng, Hua Yang, Hong Lu, Heng Liu

**Affiliations:** 1 Department of Radiology, Affiliated Hospital of Zunyi Medical University, Engineering Research Center of Intelligent Medical Imaging in Guizhou Higher Education Institutions, Zunyi, China; 2 Department of Medical Imaging, Chongqing Emergency Medical Center, Chongqing University Central Hospital, The Fourth People’s Hospital of Chongqing, Chongqing, China; 3 Department of Medical Imaging, Chongqing Traditional Chinese Medicine Hospital, Chongqing, China; 4 Department of Radiology, The Seventh People’s Hospital of Chongqing, The Central Hospital Affiliated to Chongqing University of Technology, Chongqing, China

**Keywords:** Hypertensive intracerebral hemorrhage, Hematoma expansion, Radiomics, Machine learning, Prediction model

## Abstract

**Objectives:**

This study aimed to establish a hematoma expansion (HE) prediction model for hypertensive intracerebral hemorrhage (HICH) patients by combining CT radiomics, clinical information, and conventional imaging signs.

**Methods:**

A retrospective continuous collection of HICH patients from three medical centers was divided into a training set (*n* = 555), a validation set (*n* = 239), and a test set (*n* = 77). Extract radiomics features from baseline CT plain scan images and combine them with clinical information and conventional imaging signs to construct radiomics models, clinical imaging sign models, and hybrid models, respectively. The models will be evaluated using the area under the curve (AUC), clinical decision curve analysis (DCA), net reclassification index (NRI), and integrated discrimination improvement (IDI).

**Results:**

In the training, validation, and testing sets, the radiomics model predicts an AUC of HE of 0.885, 0.827, and 0.894, respectively, while the clinical imaging sign model predicts an AUC of HE of 0.759, 0.725, and 0.765, respectively. Glasgow coma scale score at admission, first CT hematoma volume, irregular hematoma shape, and radiomics score were used to construct a hybrid model, with AUCs of 0.901, 0.838, and 0.917, respectively. The DCA shows that the hybrid model had the highest net profit rate. Compared with the radiomics model and the clinical imaging sign model, the hybrid model showed an increase in NRI and IDI.

**Conclusion:**

The hybrid model based on CT radiomics combined with clinical and radiological factors can effectively individualize the evaluation of the risk of HE in patients with HICH.

**Clinical relevance statement:**

CT radiomics combined with clinical information and conventional imaging signs can identify HICH patients with a high risk of HE and provide a basis for clinical-targeted treatment.

**Key Points:**

- *HE is an important prognostic factor in patients with HICH*.
- *The hybrid model predicted HE with training, validation, and test AUCs of 0.901, 0.838, and 0.917, respectively*.
- *This model provides a tool for a personalized clinical assessment of early HE risk*.

## Introduction

Intracerebral hemorrhage (ICH) is a prevalent cerebrovascular disorder, comprising 10–15% of all strokes [1]. Hypertensive intracerebral hemorrhage (HICH) is the most frequent form of spontaneous ICH, comprising 50–70%. Patients have a high risk of death and disability [2, 3]. Hematoma expansion (HE) is a significant factor in worsening the condition of ICH patients and causing poor outcomes, with an occurrence rate of 13–38% [4]. It has been reported that a 1 mL increase in hematoma volume is associated with a 5% (95% confidence interval (CI): 2–9%) higher risk of death or dependency. Any intervention that could reduce the size of hematoma by 2–4 mL could lead to a 10–20% decrease in the probability of mortality or disability at 90 days [5]. Therefore, accurate recognition of the possibility of HE after ICH is crucial for timely intervention and improving the prognosis of patients.

Currently, the most commonly used methods for predicting HE include assessment of imaging signs, laboratory tests, clinical characteristics, and scoring systems [6]. Our previous research on the prediction of HE in HICH patients based on the nomogram model of clinical factors and omputed tomography (CT) plain scan signs revealed that the receiver operating characteristic curve (ROC) area under the curve (AUC) being 0.762 (CI: 0.703–0.821) [7]. This conventional HE prognostication technique necessitates physicians to possess extensive clinical expertise and make subjective assumptions. Recent advances in radiomics and its application to clinical research have provided novel approaches to the quantitative analysis of HE. Ma et al [8] extracted 576 radiological features from the CT images of 254 patients with HICH. The AUC of predicting HE was 0.892, and the accuracy, sensitivity, and specificity were 0.852, 0.808, and 0.835, respectively. The research results show that radiomics can provide a reliable tool for predicting HE. Song et al [9] achieved good results by using CT radiomics and a machine learning (ML) algorithm to predict an early rise in HE with AUCs of 0.960 and 0.867 in the training and internal validation sets, respectively. Previous studies lacked the integration of radiomics with clinical and imaging features, as well as the comparison and visual display of different models.

This study aims to construct a HE prediction model based on CT plain scan radiomics and ML algorithms, combined with clinical information and conventional imaging signs, including a radiomics model, a clinical imaging sign model, and a hybrid model. The model was evaluated and validated from multiple perspectives, and displayed in the form of a nomograph, providing an intuitive and reliable guidance tool for clinical work.

## Materials and methods

### Patient selection

Our research was approved by the Ethics Committee of The Fourth People’s Hospital of Chongqing (no. 2021-62). This study was a retrospective study and was exempted from the requirement of having patients provide signed informed consent.

This study retrospectively collected 794 cases of hypertensive cerebral hemorrhage diagnosed and treated by the Chongqing Emergency Medical Center and the Affiliated Hospital of Zunyi Medical University from January 2017 to July 2023. To achieve a balance between images and data, all cases are randomly divided into a training set and a validation set at 7:3. A retrospective collection of 77 cases of HICH treated at the People’s Hospital of Guizhou Province from June 2018 to March 2023 was conducted as the test set. Each patient was selected according to the following inclusion criteria: (1) had an accurate history of hypertension (hypertension was defined as systolic pressure ≥ 140 mmHg or diastolic pressure ≥ 90 mmHg); (2) had received their first CT scan within 24 h of disease onset; (3) had completed a CT re-examination within 24 h of disease onset; and (4) had a hemorrhage that was located in the brain parenchyma, including the basal ganglia, thalamus, brain lobes, brainstem, and cerebellum.

Exclusion criteria applied for each patient were as follows: (1) had secondary ICH caused by brain trauma, cerebrovascular malformations, aneurysms, and tumors; (2) had ischemic cerebral infarction hemorrhagic transformation; (3) had undergone surgical treatment (e.g., hematoma removal or puncture drainage) before the CT examination; (4) had images of poor quality that could not be accurately evaluated; and (5) had a hematoma with borders that could not be delineated, thus precluding delineation of the region of interest (ROI).

### Collection of clinical information and conventional imaging signs

See Table 1 for clinical information and conventional imaging signs. Retrospective collection of clinical information and laboratory examinations from patients through the hospital’s case system. Clinical information is provided by neurologists who have received professional training in the diagnosis and treatment of strokes upon admission. The laboratory examination is the test result of the patient within 24 h of admission, and the laboratory examination results are divided into normal and abnormal based on whether they are within the normal range. The collection of imaging signs was independently completed by two neuroimaging attending physicians with more than seven years of work experience. The identification of signs was based on ref. [10]. When two doctors have inconsistent diagnoses of physical signs, they should refer to the literature for further discussion or seek guidance from the chief neuroimaging physician with twenty years of diagnostic work experience to reach a consensus. When the missing value of the observation index data was greater than or equal to 20%, the observation index was removed. For missing values below 20%, the multiple imputation method was employed to fill the gap [11].

**Table 1:** Patient baseline characteristics of the training set and the validation set

Category	Training set, (555 cases)	Validation set, (239 cases)	Test set, (77 cases)
HE, *n* (%)	173 (31.2)	59 (24.7)	20 (26.0)
Sex (male), *n* (%)	395 (71.2)	165 (69.0)	52 (67.5)
Age (years), mean ± SD	59.7 ± 12.7	58.8 ± 13.2	58.9 ± 13.8
Time from disease onset to first CT scan (h), median (IQR)	3.0 (3.0)	3.2 (3.2)	5.0 (8.0)
Systolic pressure at admission (mmHg), mean ± SD	170.7 ± 25.4	171.3 ± 22.9	165.9 ± 26.1
Diastolic pressure at admission (mmHg), median (IQR)	98.0 (21.0)	97.0 (21.0)	97.8 ± 16.1
GCS score at admission (scores), median (IQR)	12.0 (3.0)	12.0 (4.0)	14.0 (4.0)
White blood cell count (× 10^9^/L), median (IQR)	9.6 (5.0)	9.9 (4.7)	8.9 (5.5)
Platelet count (× 10^9^/L), median (IQR)	190.0 (75.0)	200.0 (73.5)	180.0 (90.0)
Crea (mmol/L), median (IQR)	65.2 (27.0)	67.0 (29.8)	73.0 (33.1)
Na (mmol/L), median (IQR)	139.0 (3.7)	139.2 (3.8)	140.0 (4.0)
K (mmol/L), median (IQR)	3.6 (0.5)	3.7 (0.5)	3.7 (0.6)
Ca (mmol/L), median (IQR)	2.2 (0.1)	2.2 (0.2)	2.2 (0.2)
Mg (mmol/L), median (IQR)	0.9 (0.1)	0.9 (0.1)	0.8 (0.2)
International normalized ratio, median (IQR)	1.0 (0.1)	1.0 (0.1)	1.0 (0.2)
Activated partial thromboplastin time(s), median (IQR)	31.8 (5.9)	32.3 (6.5)	27.0 (5.4)
Fibrinogen (g/L), median (IQR)	3.0 (1.0)	3.0 (1.0)	2.7 (0.9)
Diabetes mellitus history, *n* (%)	47 (8.5)	20 (8.4)	7 (9.1)
Smoking history, *n* (%)	122 (22.0)	46 (19.2)	3 (3.9)
Past cerebral stroke history, *n* (%)	53 (9.5)	21 (8.8)	5 (6.5)
First CT hematoma volume (mL), median (IQR)	16.4 (20.2)	14.1 (19.2)	11.0 (18.3)
Midline displacement (mm), median (IQR)	2.0 (4.0)	0.0 (4.2)	0.0 (0.0)
Hematoma location, *n* (%)			
Basal ganglia or thalamus	453 (81.6)	197 (82.4)	60 (77.9)
Brain lobes	31 (5.6)	12 (5.0)	9 (11.7)
Brainstem	32 (5.8)	16 (6.7)	4 (5.2)
Cerebellum	39 (7.0)	14 (5.9)	4 (5.2)
Ventricular system hemorrhage, *n* (%)	179 (32.3)	83 (34.7)	17 (22.1)
Subarachnoid hemorrhage, *n* (%)	51 (9.2)	18 (7.5)	2 (2.6)
Heterogeneous hematoma density, *n* (%)	38 (6.8)	20 (8.4)	6 (7.8)
Irregular hematoma shape, *n* (%)	189 (34.1)	78 (32.6)	17 (22.1)
Hypodensity sign, *n* (%)	140 (25.2)	49 (20.5)	19 (24.7)
Black hole sign, *n* (%)	28 (5.0)	8 (3.3)	0 (0.0)
Swirl sign, *n* (%)	217 (39.1)	90 (37.7)	25 (32.5)
Blend sign, *n* (%)	127 (22.9)	39 (16.3)	7 (9.1)
Island sign, *n* (%)	74 (13.3)	29 (12.1)	2 (2.6)
Satellite sign, *n* (%)	166 (29.9)	65 (27.2)	15 (19.5)
Fluid level sign, *n* (%)	27 (4.9)	8 (3.3)	1 (1.3)

*HE* hematoma expansion, *CT* computed tomography, *GCS* Glasgow coma scale, *Na* natrium, *K* kalium, *Ca* calcium, *Mg* magnesium, *n* number of cases, *SD* standard deviation, *IQR* interquartile range

### Hematoma volume measurement

The open-source software 3D Slicer was used to outline the hematoma layer by layer and measure its volume. The volumes of the hematoma at the first CT scan (V1) and the last CT examination within 24 h of disease onset (V2) were measured separately. Hematoma volume measurement is independently completed by the attending neuroimaging physician.

### Definition of HE and patient grouping

According to the standard explanation proposed in the literature [9], HE was defined as a V2 larger than V1 by more than 33% or an absolute increase greater than 6 mL. The patients were divided into the HE and non-HE groups.

### CT image acquisition, segmentation, and radiomics feature extraction

Image collection was performed using Lightspeed VCT 64-slice spiral CT (GE), revolution 128-slice spiral CT (GE), UCT760 64-slice spiral CT (United Imaging), and Siemens SOMATOM Force dual-source CT (Siemens) for the cranial plain CT scan. The scan range extended from the base of the skull to the top of the head, with a slice thickness of 5 mm and no slice interval. The tube voltage was set to 120 kV, and the tube current ranged from 200–238 mAs. Physician I used the open-source software 3D Slicer to delineate the ROI without knowing the patient’s clinical data and CT re-examination results. The semiautomatic layer-by-layer delineation method was adopted. The hematoma range was first delineated layer by layer using automatic recognition of the hematoma boundary by level tracing. When the hematoma boundary was delineated incorrectly, the contour was expanded and deleted manually. When a hematoma extends into the ventricles of the brain, the contours of the ventricles are used as the boundary, and only the hematoma in the brain parenchyma is used as the ROI, excluding intraventricular hemorrhage and adjacent brain parenchyma. An ROI was delineated to show the border of the hematoma and the surrounding brain tissue with a window width of 80 HU and a window level of 35 HU. Sixty patient images were randomly selected to be imported into 3D Slicer again. Physician II sketched the ROI again and then extracted the radiomics features of the ROI delineated twice. The intraclass correlation coefficient (ICC) was used to test the consistency of the two observers’ extracted radiomics features.

The Python programming language’s open-source library “PyRadiomics” (version 3.8.8) was used to extract the radiomics feature data from the segmented image. The images were preprocessed before feature extraction in Radiomics using Python’s radiomics feature extractor configuration file. This involved resampling all images to 1 × 1 × 1 mm^3^ and discretizing the grayscale data into 25 bin values using nearest neighbor interpolation. To avoid differences caused by varying acquisition methods and devices, “normalize” was set to “true” to standardize the images. A total of 1218 radiomics features were extracted, including 14 shape features, 252 first-order features, 308 gray level cooccurrence matrix (GLCM) features, 224 gray level run length matrix (GLRLM) features, 224 gray level size zone matrix (GLSZM) features, and 196 gray level dependence matrix (GLDM) features.

### Radiomics feature selection and radiomics model construction

When the ICC was > 0.75, the features were assessed as having high stability and repeatability, and were retained [12]. The features of the training set were standardized using the *Z*-score, and the same normalization process with the mean and standard deviation values of the training set was applied to the validation and testing sets. The radiomics features in the training set were selected, and eight different ML algorithms were used to train the model. To prevent overfitting of the model, a 10-fold cross-validation method was used to validate the algorithm and select the most stable ML algorithm model. The steps were as follows: (1) a *t*-test was used to screen out the radiomics features with a statistically significant difference (*p* < 0.05) between the HE group and the non-HE group; (2) least absolute shrinkage and selection operator (LASSO) regression method based on 10-fold cross-validation for further feature screening, penalty coefficient (λ) based on the minimum binomial deviation plus one standard deviation; (3) redundant features with a correlation coefficient *r* > 0.8 were eliminated by Pearson correlation analysis among the radiomics features; (4) the final selected radiomics features were used to construct a prediction model in the training set using logistic regression (LR), k-nearest neighbors (KNN), support vector machines (SVM), decision trees (DT), random forests (RF), linear discriminant analysis (LDA), quadratic discriminant analysis (QDA), and naive bayes (NB) algorithms; (5) Select the most stable ML algorithm using 10-fold cross-validation; and (6) the most stable ML algorithm was used to construct a radiomics model and calculate the radiomics score (Rad score), Rad score = ln(*p*/(1 − *p*)), where *p* represents the probability of the occurrence of HE predicted by a radiomics model in each patient.

### Statistical analysis

Statistical analyses were performed using SPSS (version 26.0) and R software (version 4.0.0). The independent risk factors for HE in clinical information and conventional imaging signs were screened by univariate and multivariate LR analysis (*p* < 0.05), a clinical imaging sign model was constructed, and a hybrid model was built with a Rad score. The discrimination of the ROC curve and the AUC evaluation model, the calibration degree of the Hosmer Lemeshow test and the calibration curve evaluation model, and the practicability of the clinical decision curve analysis (DCA) evaluation model. The model’s diagnostic accuracy, sensitivity, specificity, positive predictive value (PPV), and negative predictive value (NPV), net reclassification index (NRI), and integrated discrimination improvement (IDI) were calculated.

## Results

### Patient characteristics

**Fig. 1:**
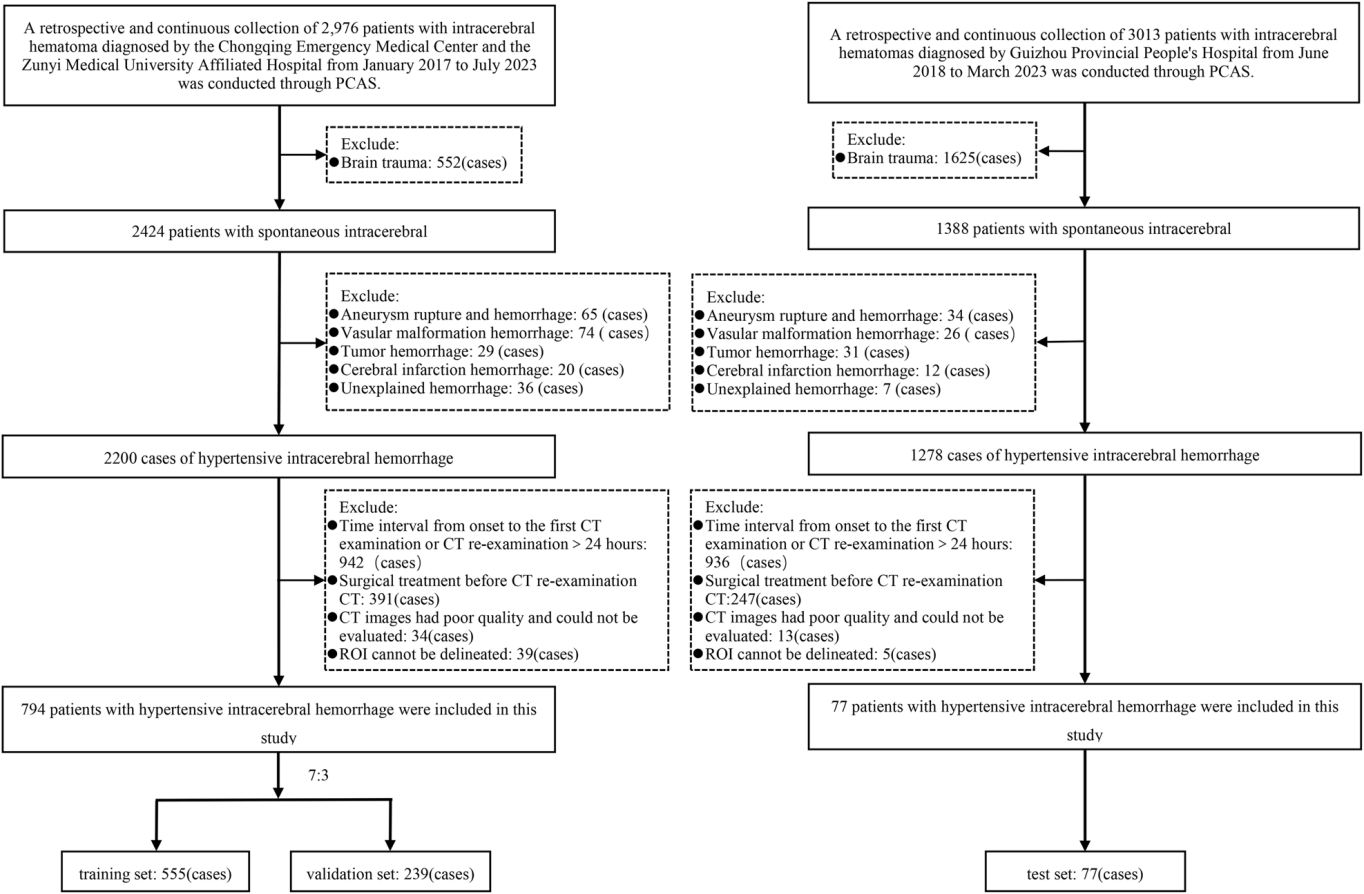
The flow chart for patient screening. PACS, picture archiving and communication systems

A total of 555 patients were included in the training set, 239 patients were included in the validation set, and 77 patients were included in the test set. The patient screening flow chart is shown in Fig. 1. Table 1 provides clinical information and conventional imaging sign data.

### Radiomics model

**Fig. 2:**
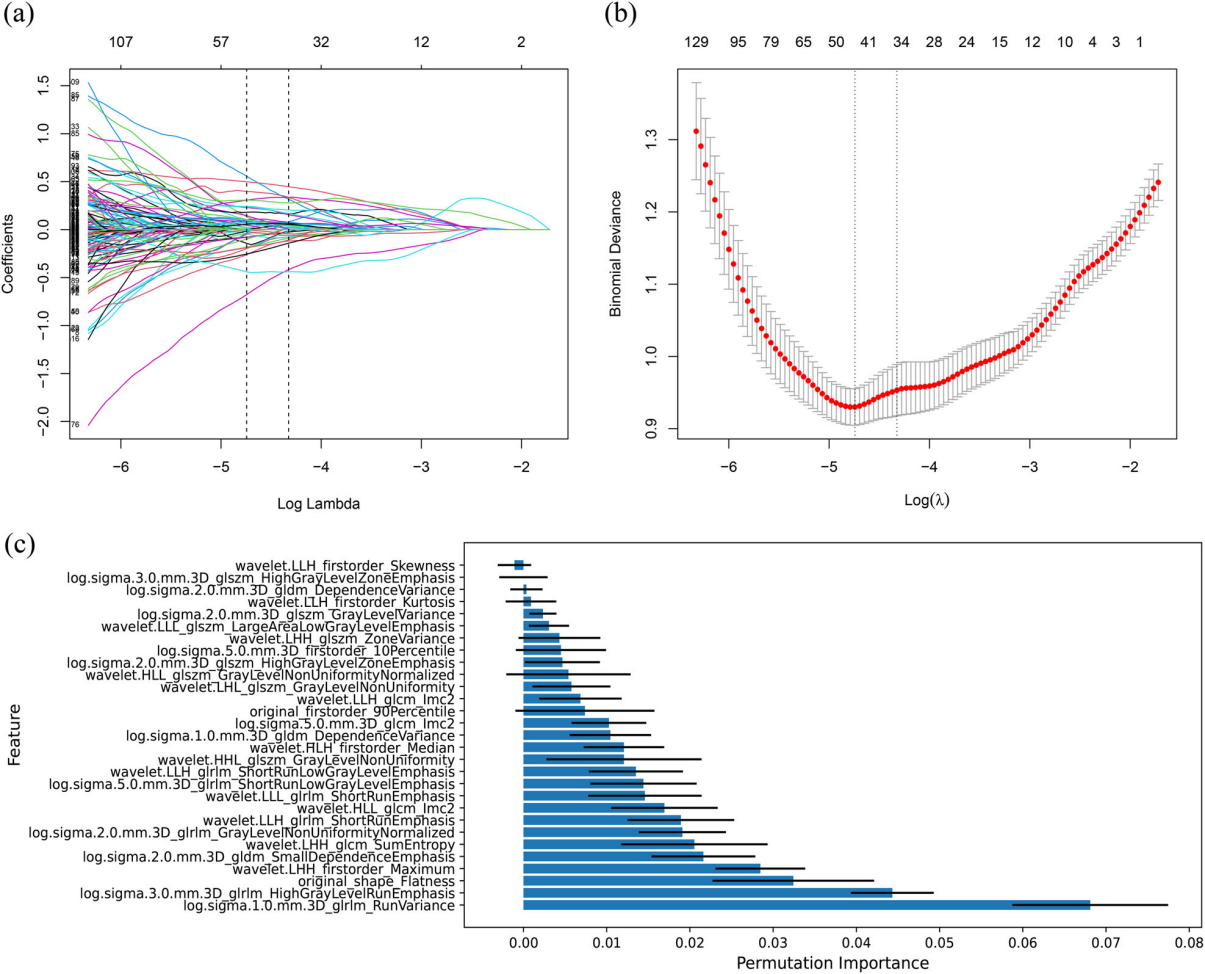
The figure illustrates the process of radiomics feature selection. **a** The feature selection of LASSO regression is based on the minimum binomial deviation of the model plus one standard error to review features with non-zero coefficients. **b** The 10-fold cross-validation process for LASSO regression feature selection is based on the minimum binomial deviation of the model plus one standard error, multiplied by the logarithm of λ. This value is equal to −4.325. **c** Proving the importance of specific radiomics features through histograms

One thousand nineteen radiomics characteristics ICC > 0.75. 624 radiomics features had statistically significant differences (*p* < 0.05) between the HE and non-HE groups in the training set. Through the 10-fold cross-validation LASSO regression method, 36 radiomics features were selected (Fig. 2a, b). Seven redundant features with a Pearson correlation coefficient *r* > 0.8 were removed, and 29 radiomics features were screened. See Fig. 2c for the importance histogram of each feature. LR, KNN, SVM, DT, RF, LDA, QDA, and NB ML algorithms have AUCs of 0.885, 0.864, 0.860, 0.839, 1.000, 0.875, 0.869, and 0.788, respectively, in the training set. The average AUC for 10-fold cross-validation was 0.845, 0.791, 0.844, 0.645, 0.793, 0.840, 0.754, and 0.768, respectively. Choose the LR algorithm as the most stable to construct a radiomics model. In the training set, the radiomics model predicted an AUC of 0.885 (95% CI: 0.856–0.914, *p* < 0.001) for HE. In the validation set, the model predicted an AUC of 0.827 (95% CI: 0.760–0.893, *p* < 0.001) for HE. In the test set, the model predicted an AUC of 0.894 (95% CI: 0.793–0.995, *p* < 0.001) for HE. The *p* values of the Hosmer–Lemeshow test in the training set, the validation set, and the test set were 0.725 (χ^2^ = 5.305), 0.247 (χ^2^ = 10.258), and 0.270 (χ^2^ = 9.931), respectively (all *p* > 0.05).

### Clinical imaging sign model

The training set was subjected to both univariate and multivariate LR analyses based on the clinical information and conventional imaging signs, and the results are presented in Table 2. Time from disease onset to first CT scan, GCS score at admission, smoking history, first CT hematoma volume, irregular hematoma shape, and blend sign are independent predictors of HE to build a clinical imaging sign model. The model predicts an AUC of 0.759 (95% CI: 0.714–0.804, *p* < 0.001) for predicting HE in the training set, 0.725 (95% CI: 0.647–0.804, *p* < 0.001) in the validation set, and 0.765 (95% CI: 0.631–0.898, *p* < 0.001) in the test set. The *p* values of the Hosmer–Lemeshow test in the training set, validation set, and test set were 0.493 (χ^2^ = 7.408), 0.307 (χ^2^ = 9.438), and 0.265 (χ^2^ = 10.005), respectively (all *p* > 0.05).

**Table 2:** The LR results, both univariate and multivariate, using clinical information and routine imaging signs were obtained

	Univariate		Multivariate	
	OR, (95% CI)	*p*	OR, (95% CI)	*p*
Sex	0.52 (0.34–0.80)	0.003^*^	0.72 (0.43–1.20)	0.205
Age	0.99 (0.98–1.01)	0.416	–	–
Time from disease onset to first CT scan	0.92 (0.85–0.98)	0.017^*^	0.92 (0.84–1.00)	0.041^*^
Systolic pressure at admission	1.01 (1.00–1.01)	0.068	–	–
Diastolic pressure at admission	1.01 (1.00–1.02)	0.114	–	–
GCS score at admission	0.79 (0.74–0.86)	< 0.001^*^	0.86 (0.79–0.94)	< 0.001^*^
White blood cell count	1.44 (1.00–2.07)	0.048^*^	1.36 (0.90–2.08)	0.148
Platelet count	1.33 (0.78–2.26)	0.289	–	–
Crea	1.25 (0.83–1.87)	0.291	–	–
Na	0.90 (0.57–1.42)	0.648	–	–
K	1.27 (0.87–1.85)	0.219	–	–
Ca	1.32 (0.85–2.07)	0.218	–	–
Mg	1.15 (0.70–1.87)	0.579	–	–
International normalized ratio	0.79 (0.48–1.29)	0.342	–	–
Activated partial thromboplastin time	0.73 (0.50–1.08)	0.113	–	–
Fibrinogen (g/L)	1.46 (0.67–3.18)	0.344	–	–
Diabetes mellitus history	0.74 (0.37–1.46)	0.385	–	–
Smoking history	1.83 (1.21–2.78)	0.004^*^	1.73 (1.04–2.87)	0.034^*^
Past cerebral stroke history	0.95 (0.51–1.76)	0.871	–	–
First CT hematoma volume	1.04 (1.03–1.05)	< 0.001^*^	1.02 (1.01–1.04)	0.008^*^
Midline displacement	1.18 (1.11–1.27)	< 0.001^*^	0.97 (0.88–1.07)	0.507
Hematoma location	1.06 (0.87–1.30)	0.538		
Ventricular system hemorrhage	1.01 (0.69–1.48)	0.968	–	–
Subarachnoid hemorrhage	1.77 (0.99–3.19)	0.055	–	–
Heterogeneous hematoma density	2.64 (1.36–5.14)	0.004^*^	0.96 (0.42–2.22)	0.929
Irregular hematoma shape	4.18 (2.85–6.12)	< 0.001^*^	3.14 (1.87–5.29)	< 0.001^*^
Hypodensity sign	2.57 (1.72–3.82)	< 0.001^*^	1.61 (0.89–2.90)	0.112
Black hole sign	2.31 (1.08–4.97)	0.031^*^	0.96 (0.38–2.40)	0.924
Swirl sign	2.10 (1.46–3.03)	< 0.001^*^	1.04 (0.64–1.70)	0.863
Blend sign	2.39 (1.59–3.60)	< 0.001^*^	1.87 (1.15–3.05)	0.012^*^
Island sign	2.23 (1.36–3.67)	0.002^*^	0.83 (0.40–1.72)	0.618
Satellite sign	1.95 (1.33–2.86)	< 0.001^*^	0.62 (0.34–1.12)	0.113
Fluid level sign	2.93 (1.34–6.40)	0.007^*^	1.68 (0.66–4.25)	0.278

*CT* computed tomography, *GCS* Glasgow coma scale, *Na* natrium, *K* kalium, *Ca* calcium, *Mg* magnesium, *OR* odds ratio, *CI* confidence interval

* *p* < 0.05, indicating that the difference was statistically significant

### Hybrid model

Time from disease onset to first CT scan, GCS score at admission, smoking history, first CT hematoma volume, irregular hematoma shape, blend sign, and Rad score were included in multivariate LR analysis, excluding variables with small contributions of time from disease onset to first CT scan (OR: 0.913, 95% CI: 0.826–1.002, *p* = 0.064), blend sign (OR: 1.697, 95% CI: 0.932–3.091, *p* = 0.083), and smoking history (OR: 1.607, 95% CI: 0.895–2.883, *p* = 0.111) to the model. Finally, the GCS score at admission, first CT hematoma volume, irregular hematoma shape, and Rad score were chosen to build the hybrid model. (see Table 3). The model predicts an AUC of 0.901 (95% CI: 0.874–0.927, *p* < 0.001) for predicting HE in the training set, 0.838 (95% CI: 0.773–0.902, *p* < 0.001) in the validation set, and 0.917 (95% CI: 0.824–1.000, *p* < 0.001) in the test set. The *p* values of the Hosmer–Lemeshow test in the training set and the validation set were 0.746 (χ^2^ = 5.110), 0.142 (χ^2^ = 12.220), and 0.501 (χ^2^ = 7.333), respectively (all *p* > 0.05).

**Table 3:** Multivariate LR analysis results of a hybrid model

Factor	*Β* value	SE	*Z* value	*p*	OR (95% CI)
GCS score at admission	−0.112	0.051	−2.191	0.028^*^	0.894 (0.808–0.988)
First CT hematoma volume	−0.018	0.009	−1.997	0.046^*^	0.982 (0.965–0.999)
Irregular hematoma shape	1.211	0.291	4.160	< 0.001^*^	3.357 (1.906–5.982)
Rad score	0.999	0.100	9.978	< 0.001^*^	2.715 (2.255–3.341)
Constant	1.182	0.649	1.820	0.068	3.261 (0.919–11.839)

*GCS* Glasgow coma scale, *CT* computed tomography, *Rad score* radiomics score, *SE* standard error, *OR* odds ratio, *CI* confidence interval

* *p* < 0.05, indicating that the difference was statistically significant

### Evaluation of the model’s prediction effectiveness and its display

**Fig. 3:**
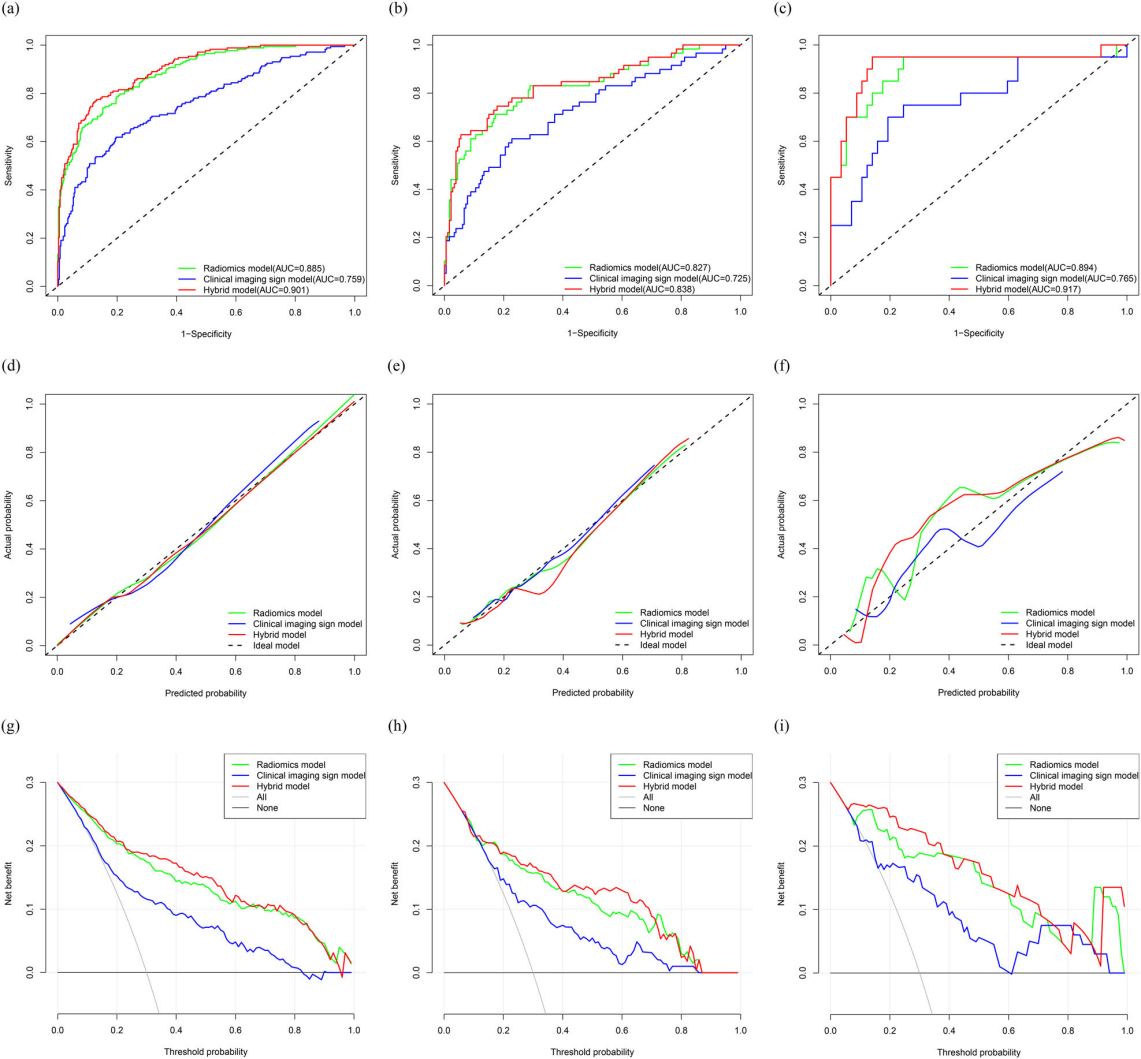
ROC curve, calibration curve, and clinical decision curve. **a** ROC curve of the training set; **b** ROC curve of the validation set; **c** ROC curve of the test set; **d** the training set calibration curve; **e** validation set calibration curve; **f** test set calibration curve; **g** training set clinical decision curve; **h** validation set clinical decision curve; and (**i**) test set clinical decision curve

**Fig. 4:**
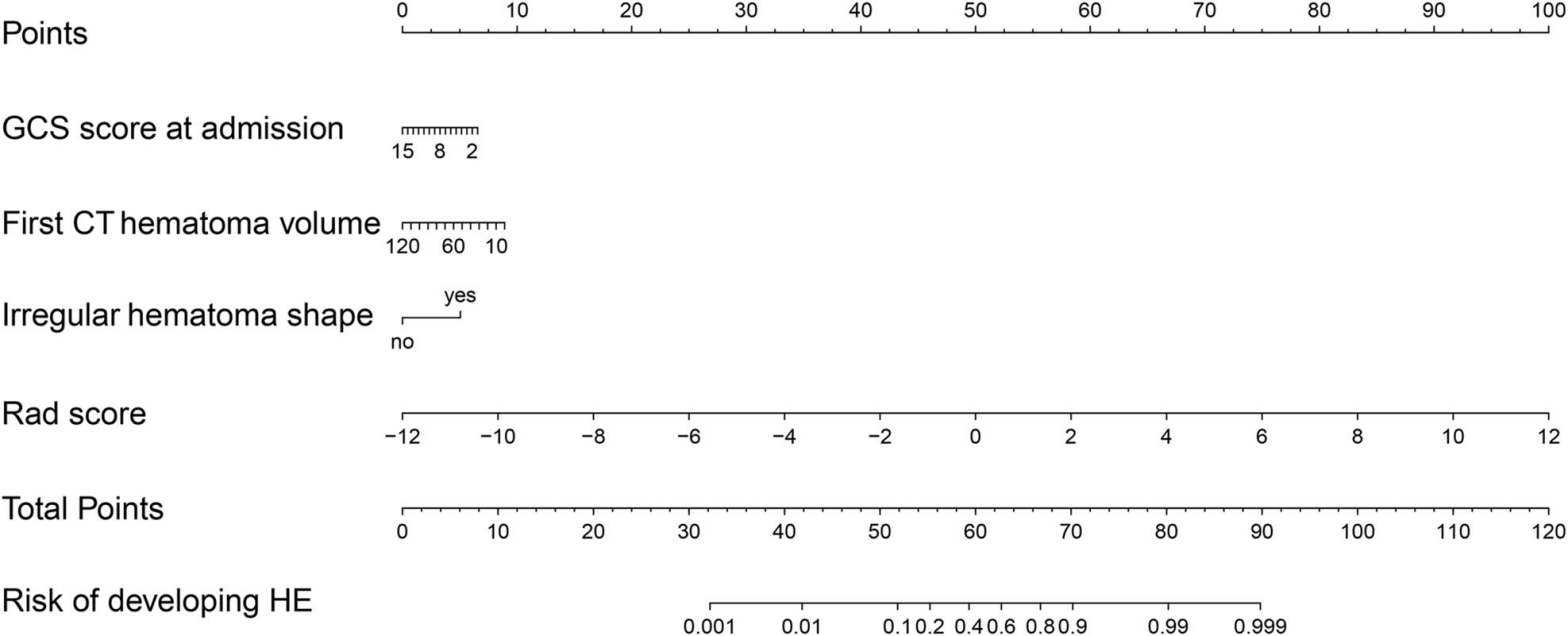
Nomogram of hybrid model prediction HE. GCS, Glasgow coma scale; Rad score, radiomics score; HE, hematoma expansion

Balance the sensitivity, specificity, and accuracy of the model by using the best cutoff value from the grid search (with a search interval of 0.10–0.50 and a search interval of 0.05). The search found that when the cutoff values for the radiomics model, clinical imaging sign model, and hybrid model were set to 0.25, each model had the best sensitivity, specificity, and accuracy. The accuracy, sensitivity, specificity, PPV, and NPV of the training set, validation set, and test set of the three models were calculated; see Table 4 for the results. The AUC of the hybrid model was the largest in both the training set, the validation set, and the test set (Fig. 3a–c). The calibration curves in Fig. 3d–f revealed that the expected probability values of the three models in the training set, validation set, and test set were consistent with the real probability values. The clinical decision curve (Fig. 3g–i) revealed that the hybrid model had the broadest domain probability range and the highest clinical net benefit rate in both the training set, validation set, and test set. In the training set, the NRI and IDI of the hybrid model increased by approximately 0.499 (95% CI: 0.325–0.673, *p* < 0.001) and 0.039 (95% CI: 0.022–0.057, *p* < 0.001), respectively, compared to the radiomics model. The hybrid model increased by approximately 1.098 (95% CI: 0.947–1.248, *p* < 0.001) and 0.265 (95% CI: 0.220–0.311, *p* < 0.001) compared to the clinical imaging sign model, respectively. In the validation set, the NRI and IDI of the hybrid model increased by approximately 0.593 (95% CI: 0.309–0.876, *p* < 0.001) and 0.036 (95% CI: 0.011–0.061, *p* = 0.006), respectively, compared to the radiomics model. The hybrid model increased by approximately 0.790 (95% CI: 0.522–1.059, *p* < 0.001) and 0.252 (95% CI: 0.164–0.340, *p* < 0.001) compared to the clinical imaging sign model, respectively. In the test set, the NRI and IDI of the hybrid model increased by approximately 0.849 (95% CI: 0.377–1.322, *p* < 0.001) and 0.033 (95% CI: −0.015 to 0.080, *p* = 0.179), respectively, compared to the radiomics model. The hybrid model increased by approximately 0.949 (95% CI: 0.487–1.411, *p* < 0.001) and 0.329 (95% CI: 0.169–0.488, *p* < 0.001) compared to the clinical imaging sign model, respectively. Based on the prediction effectiveness evaluation of the model, a nomograph was drawn with a hybrid model as shown in Fig. 4. In the nomograph, the score of each independent predictor is the points corresponding to the upper scoring scale, and the total point of each patient is the sum of the scores of each independent predictor. The value of the total score corresponding to the risk axis of HE occurrence is the risk of HE occurrence. The higher the total score, the greater the risk. Based on the diversity of the patient population, we conducted subgroup analysis on the hybrid model in the validation and testing sets (Table 5), including gender (male, female), patient age (≤ 65 years, 66–79 years, and ≥ 80 years), time from onset to first CT scan (< 3 h, 3–6 h, and > 6 h), first CT hematoma volume (< 15 mL, 15–30 mL, and > 30 mL), GCS score at admission (3–8 points, 9–11 points, 12–14 points, and 15 points), and hematoma location (basal ganglia and thalamus, lobes, brainstem, and cerebellum).

**Table 4:** The model predicts the effectiveness of HE

Model	AUC (95% CI)	Accuracy	Sensitivity	Specificity	PPV	NPV
Training set						
Radiomics model	0.885 (0.856–0.914)	0.757	0.827	0.725	0.576	0.902
Clinical imaging sign model	0.759 (0.714–0.804)	0.656	0.717	0.628	0.466	0.830
Hybrid model	0.901 (0.874–0.927)	0.780	0.821	0.762	0.609	0.904
Validation set						
Radiomics model	0.827 (0.760–0.893)	0.736	0.780	0.722	0.479	0.909
Clinical imaging sign model	0.725 (0.647–0.804)	0.649	0.678	0.639	0.381	0.858
Hybrid model	0.838 (0.773–0.902)	0.724	0.796	0.700	0.465	0.913
Test set						
Radiomics model	0.894 (0.793–0.995)	0.870	0.700	0.930	0.778	0.898
Clinical imaging sign model	0.765 (0.631–0.898)	0.766	0.600	0.825	0.546	0.855
Hybrid model	0.917 (0.824–1.000)	0.883	0.850	0.894	0.739	0.944

*HE* hematoma expansion, *AUC* area under the receiver operating characteristic curve, *CI* confidence interval, *PPV* positive predictive value, *NPV* negative predictive value

**Table 5:** Subgroup analysis of the hybrid model in the validation and testing sets

	*N*, (%)	AUC, (95% CI)	Accuracy	Sensitivity	Specificity
Validation set					
Sex					
Male	165 (69.0%)	0.858 (0.786–0.931)	0.773	0.814	0.705
Female	74 (31.0%)	0.783 (0.650–0.917)	0.703	0.750	0.690
Age, (years)					
≦ 65	168 (70.3%)	0.867 (0.798–0.936)	0.744	0.837	0.712
66–79	53 (22.2%)	0.721 (0.553–0.908)	0.698	0.636	0.714
≧ 80	18 (7.5%)	0.831 (0.598–1.000)	0.611	0.800	0.539
Time from disease onset to first CT scan (h)					
< 3	93 (38.9%)	0.839 (0.740–0.938)	0.742	0.833	0.710
3–6	107 (44.8%)	0.805 (0.700–0.909)	0.636	0.724	0.641
> 6	39 (16.3%)	0.939 (0.861–1.000)	0.846	1.000	0.818
First CT hematoma volume (mL)					
< 15	126 (52.7%)	0.850 (0.741–0.959)	0.794	0.778	0.796
15–30	63 (26.4%)	0.741 (0.573–0.909)	0.714	0.625	0.745
> 30	50 (20.9%)	0.736 (0.596–0.876)	0.560	0.920	0.200
GCS score at admission (scores)					
3–8	30 (12.6%)	0.824 (0.656–0.992)	0.600	0.833	0.444
9–11	79 (33.1%)	0.864 (0.774–0.955)	0.646	0.917	0.527
12–14	97 (40.6%)	0.732 (0.593–0.872)	0.724	0.600	0.779
15	33 (13.8%)	1.000 (1.000–1.000)	0.970	1.000	0.967
Hematoma location					
Basal ganglia or thalamus	197 (82.4)	0.861 (0.794–0.928)	0.736	0.833	0.705
Brain lobes	12 (5.0)	0.594 (0.253–0.935)	0.500	0.500	0.500
Brainstem	16 (6.7)	0.850 (0.605–1.000)	0.625	0.833	0.500
Cerebellum	14 (5.9)	0.538 (0.267–0.809)	0.857	0.000	0.923
Test set					
Sex					
Male	52 (67.5%)	0.890 (0.771–1.000)	0.865	0.800	0.892
Female	25 (32.5%)	0.990 (0.959–1.000)	0.920	1.000	0.900
Age (years)					
≦ 65	52 (67.5%)	0.869 (0.718–1.000)	0.827	0.727	0.854
66–79	19 (24.7%)	1.000 (1.000–1.000)	1.000	1.000	1.000
≧ 80	6 (7.8%)	1.000 (1.000–1.000)	1.000	1.000	1.000
Time from disease onset to first CT scan (h)					
< 3	24 (31.2%)	1.000 (1.000–1.000)	0.958	1.000	0.944
3–6	24 (31.2%)	0.852 (0.635–1.000)	0.875	0.750	0.938
> 6	29 (37.6%)	0.899 (0.783–1.000)	0.827	0.833	0.826
First CT hematoma volume (mL)					
< 15	47 (61.0%)	0.875 (0.647–1.000)	0.979	0.857	1.000
15–30	20 (26.0%)	0.786 (0.580–0.991)	0.650	0.667	0.643
> 30	10 (13.0%)	0.905 (0.695–1.000)	0.900	1.000	0.667
GCS score at admission (scores)					
3–8	7 (9.1%)	0.800 (0.449–1.000)	0.857	0.800	1.000
9–11	15 (19.5%)	0.981 (0.925–1.000)	0.867	1.000	0.778
12–14	25 (32.4%)	0.930 (0.826–1.000)	0.840	0.667	0.895
15	30 (39.0%)	0.975 (0.917–1.000)	0.933	1.000	0.926
Hematoma location					
Basal ganglia or thalamus	60 (77.9%)	0.960 (0.916–1.000)	0.883	0.833	0.896
Brain lobes	9 (11.7%)	0.750 (0.392–1.000)	0.750	0.800	0.667
Brainstem	4 (5.2%)	1.000 (1.000–1.000)	1.000	1.000	1.000
Cerebellum	4 (5.2%)	1.000 (1.000–1.000)	1.000	1.000	1.000

*N* number of cases, *GCS* Glasgow coma scale, *AUC* the area under the receiver operating characteristic curve, *CI* confidence interval

## Discussion

In this study, CT radiomics combined with clinical information and conventional imaging signs was used to predict the risk of HE in HICH patients within 24 h, and a multi-angle evaluation was conducted. The model is presented in the form of a nomogram, providing a reliable tool for clinicians to evaluate the HE risk of HICH patients.

Our research focuses on patients with HICH. Using the first CT plain scan image within 24 h of onset, we delineate the ROI, extract radiomics features, and combine them with clinical imaging signs to construct an HE prediction model. This model is integrated into the routine clinical workflow, which can quickly screen high-risk patients for HE, facilitating timely clinical treatment and improving patient prognosis. The management of patients has the following clinical practical significance: firstly, personalized treatment, helping doctors develop personalized treatment plans, predicting the risk of HE through the model, early detection of high-risk patients, early control of blood pressure, intracranial pressure monitoring, or necessary surgical treatment [13], strengthening monitoring and management of patients, preventing deterioration of patient neurological function, improving treatment effectiveness, survival rate, and quality of life. Secondly, resource optimization involves predicting the risk of HE in patients through the model, allocating appropriate medical resources to patients with different risks, and improving the efficiency and quality of medical services.

Much clinical information and conventional imaging signs have been proven to be independent risk factors for HE [14–16]. Our previous study revealed that HE had a moderate degree of differentiation based on clinical factors and CT plain-scan signs (AUC = 0.762, 95% CI: 0.703–0.821) [7]. In this study, a multivariate LR analysis revealed that time from disease onset to first CT scan, GCS score at admission, smoking history, first CT hematoma volume, irregular hematoma shape, and blend sign were independent predictors of HE and allowed a clinical imaging sign model to be built. The AUCs in the training set, validation set, and testing set were 0.759, 0.725, and 0.765, respectively, which is consistent with our previous research results. Previous studies have shown that HE after cerebral hemorrhage often occurs within the first 6 h [17]. The shorter the time from onset to the first CT scan, the greater the instability of hematoma bleeding, and the higher the probability of HE during follow-up. The GCS score reflects the patient’s consciousness from the eye-opening response, language response, and limb movement and indirectly reflects the patient’s brain tissue damage [18]. This study found that smoking is a risk factor for the occurrence of HE. Wei [19] et al found that smoking patients have a higher risk of HE (OR: 2.06; 95% CI: 1.10–3.86), which is consistent with the results of this study. In this study, the smaller the volume of hematoma on the first CT scan, the higher the risk of an increase in hematoma volume during follow-up examination. This may be due to the early stage of bleeding on the first CT scan. Over time, the bleeding continues, leading to an increase in the hematoma volume found on the follow-up CT scan. The irregular hematoma shape is characterized by three or more lobulated or island signs in the hematoma, indicating damage to the surrounding brain tissue and resulting bleeding. Barras et al found that an irregular hematoma morphology is an independent predictor of HE [20–22]. The presence of a blend sign indicates active bleeding within the hematoma and heterogeneity in the hematoma’s density [23]. Evaluation with these traditional HE prediction methods based on clinical information and conventional imaging signs requires doctors to possess a certain level of clinical experience and subjectivity.

Radiomics allows the extraction of deep-level feature information that cannot be recognized by the naked eye, thus reflecting the heterogeneity of lesions [24]. It has been demonstrated through multiple studies that radiomics can be a valuable asset in determining the diagnosis, treatment, and prognosis of different diseases [25, 26]. The study employed 1218 radiomics features extracted from segmented images. After dimensionality reduction, 29 radiomics features were selected for modeling, comprising 1 shape feature, 6 first-order features, 4 GLCM features, 3 GLDM features, 8 GLSZM features, and 7 GLRLM features. Shape features reflect the shape differences of a hematoma. First-order features reflect the heterogeneity of hematoma density by describing voxel intensity distribution and the degree of internal variability. GLCM, GLDM, GLSZM, and GLRLM features reflect the heterogeneity of hematoma density by describing the distinctive texture features of the hematoma [27]. Previous research [28] has demonstrated that the degree of heterogeneity in hematomas may indicate HE, likely reflecting the presence of ongoing hemorrhage. In our study, eight different machine-learning algorithms were used to construct a radiomics model. To prevent overfitting, ten-fold cross-validation was used to evaluate model performance and select a stable ML algorithm. The model was considered overfitted if its AUC for the training set was significantly higher than that for the validation set. Finally, using the best ML classifier, we established a radiomics model on the entire training set and calculated the Rad score using the logit function [27]. In our study, the training set AUCs for LR, KNN, SVM, DT, RF, LDA, QDA, and NB ML algorithms are 0.885, 0.864, 0.860, 0.839, 1.000, 0.875, 0.869, and 0.788, respectively. The average AUCs for 10-fold cross-validation were 0.845, 0.791, 0.844, 0.645, 0.793, 0.840, 0.754, and 0.768, respectively. Among them, the LR, SVM, and LDA algorithms are the most stable. While ensuring that the model predicts HE with a high AUC, we chose to use the LR algorithm as the optimal ML classifier. We established a radiomics model on the entire training set and calculated the Rad score using the logit function. Our research results showed that the AUC of the radiomics model and the hybrid model in the training set was 0.885 and 0.901, respectively. The AUC of the radiomics model and the hybrid model in the validation set was 0.827 and 0.838, respectively. The AUC of the radiomics model and the hybrid model in the test set was 0.894 and 0.917, respectively. Based on the above steps and methods, there was no overfitting of the model.

Recent research reports have shown that radiomics, clinical, and imaging features based on plain CT scan images can predict early HE. The models used in these studies have good predictive performance. However, few studies have focused on combining the advantages of different variables, including clinical and imaging features, radiomics, and hybrid models, to conduct in-depth evaluations and comparisons of different models. Additionally, there is a lack of large-scale and multicenter research and evaluation. For example, Pszczolkowski et al [29] conducted a study based on CT radiomics to predict early HE and poor outcomes in patients with cerebral hemorrhage. Their AUC for predicting HE in the test set was 0.69, with a sensitivity of 0.635. Compared to this study, the AUCs (0.838 and 0.917, respectively) and sensitivities (0.796 and 0.850, respectively) of the validation and testing sets were improved. Feng et al [30] conducted an early HE study based on 231 cases of spontaneous ICH using non-contrast-enhanced CT and deep-learning radiomics feature prediction. Their nomogram model predicted HE with an AUC of 0.82 and 0.83 in the internal and external validation cohorts, respectively, with a sensitivity of 0.70 and 0.54. However, this study was a multicenter study involving three independent medical institutions with a total sample size of 871. It incorporated more clinical information and constructed a clinical imaging sign model, a radiomics model, and a hybrid model to evaluate the AUC, accuracy, sensitivity, and specificity of the models from multiple perspectives. The performance of different models was thoroughly evaluated using NRI and IDI.

This study had some limitations. Firstly, there may be data selection bias in this retrospective study. Although it was a multicenter study and the model was externally validated, data collection was conducted by professional neurologists and neuroimaging physicians to minimize bias. However, in order to provide more reliable models for clinical use, further prospective analysis is necessary in the future. Secondly, there is a potential for ROI delineation bias. Inconsistent delineation of ROIs may lead to unstable extracted features. We employed two neuroimaging physicians to independently outline the ROIs and calculate the ICC, selecting features with an ICC of 0.75 or higher to reduce the bias. In the future, we must adopt an automatic and accurate hematoma segmentation method. This will not only improve the efficiency of quantitative imaging analysis, but also ensure the stability and consistency of the analysis process. Thirdly, Model deployment deviation. Although our study used multicenter studies for sufficient validation and testing to improve the model’s generalization ability, the sample size in our external test set was relatively small. Differences in regions and CT equipment may lead to a decrease in the model’s performance. In the future, prospective multicenter studies are needed to monitor the model’s performance and update it in a timely manner to ensure its effectiveness and generalizability in practical applications.

## Conclusion

The hybrid model, based on CT radiomics combined with clinical information and conventional imaging signs, can be used to effectively predict early HE in HICH. The performance of predicting HE is further improved compared to the radiomics model and the clinical imaging sign model. This model is displayed in the form of a nomograph, providing an intuitive and reliable guidance tool for a personalized clinical assessment of early HE risk in patients.

## Abbreviations

AUC: Area under the curve
CI: Confidence interval
CT: Computed tomography
DCA: Decision curve analysis
DT: Decision tree
GCS: Glasgow coma scale
GLCM: Gray level cooccurrence matrix
GLDM: Gray level dependence matrix
GLRLM: Gray level run length matrix
GLSZM: Gray level size zone matrix
HE: Hematoma expansion
HICH: Hypertensive intracerebral hemorrhage
ICC: Intraclass correlation coefficient
ICH: Intracerebral hemorrhage
IDI: Integrated discrimination improvement
KNN: k-Nearest neighbor
LASSO: Least absolute shrinkage and selection operator
LDA: Linear discriminant analysis
LR: Logistic regression
ML: Machine learning
NB: Naive bayes
NPV: Negative predictive value
NRI: Net reclassification index
PPV: Positive predictive value
QDA: Quadratic discriminant analysis
Rad score: Radiomics score
RF: Random forest
ROC: Receiver operating characteristic
ROI: Region of interest
SVM: Support vector machines
